# Podoplanin^+^ tumor lymphatics are rate limiting for breast cancer metastasis

**DOI:** 10.1371/journal.pbio.2005907

**Published:** 2018-12-28

**Authors:** Yang Chen, Doruk Keskin, Hikaru Sugimoto, Keizo Kanasaki, Patricia E. Phillips, Lauren Bizarro, Arlene Sharpe, Valerie S. LeBleu, Raghu Kalluri

**Affiliations:** 1 Department of Cancer Biology, Metastasis Research Center, University of Texas MD Anderson Cancer Center, Houston, Texas, United States of America; 2 Division of Matrix Biology, Beth Israel Deaconess Medical Center and Harvard Medical School, Boston, Massachusetts, United States of America; 3 Department of Microbiology and Immunobiology, Harvard Medical School, Boston, Massachusetts, United States of America; Friedrich Miescher Institute for Biomedical Research, University of Basel, Switzerland

## Abstract

Metastatic dissemination employs both the blood and lymphatic vascular systems. Solid tumors dynamically remodel and generate both vessel types during cancer progression. Lymphatic vessel invasion and cancer cells in the tumor-draining lymph nodes (LNs) are prognostic markers for breast cancer metastasis and patient outcome, and tumor-induced lymphangiogenesis likely influences metastasis. Deregulated tumor tissue fluid homeostasis and immune trafficking associated with tumor lymphangiogenesis may contribute to metastatic spreading; however, the precise functional characterization of lymphatic endothelial cells (LECs) in tumors is challenged by the lack of specific reagents to decipher their rate-limiting role in metastasis. Therefore, we generated novel transgenic mice (PDPN promoter-driven Cre recombinase transgene [PDPN-Cre] and PDPN promoter-driven thymidine kinase transgene [PDPN-tk]) that allow for the identification and genetically controlled depletion of proliferating podoplanin (*Pdpn*)-expressing LECs. We demonstrate that suppression of lymphangiogenesis is successfully achieved in lymphangioma lesions induced in the PDPN-tk mice. In multiple metastatic breast cancer mouse models, we identified distinct roles for LECs in primary and metastatic tumors. Our findings support the functional contribution of primary tumor lymphangiogenesis in controlling metastasis to axillary LNs and lung parenchyma. Reduced lymphatic vessel density enhanced primary tumor lymphedema and increased the frequency of intratumoral macrophages but was not associated with a significant impact on primary tumor growth despite a marked reduction in metastatic dissemination. Our findings identify the rate-limiting contribution of the breast tumor lymphatic vessels for lung metastasis.

## Author summary

Cancer progression and metastasis of solid tumors can occur in association with the generation of new lymphatic vessels (lymphangiogenesis). Lymphatic vessel invasion and cancer cells in the tumor-draining lymph nodes are used as prognostic markers for breast cancer metastasis and patient outcome. However, the specific role of newly formed lymphatic vessels in breast cancer metastasis to the lung remains unknown. In this study, we have analyzed this process by generating novel transgenic mice that enabled the identification of podoplanin (*Pdpn*)-expressing lymphatic endothelial cells, as well as the controlled depletion of these cells during lymphangiogenesis in breast cancer progression. We show that in multiple metastatic breast cancer mouse models, the specific suppression of lymphangiogenesis, without impacting blood vessel formation (angiogenesis), does not limit primary tumor growth but reduces cancer cell dissemination to the lung and metastatic disease. We conclude that inhibition of breast tumor lymphangiogenesis decreases lung metastasis without affecting primary tumor growth.

## Introduction

Metastasis is responsible for 90% of deaths of breast cancer patients [1,2]. The contribution of both cancer cells and stromal cells (such as fibroblasts, endothelial cells, pericytes, and immune cells) is important for cancer development and metastasis, including in breast cancer [2–5]. The lymphatic system, consisting of lymphatic vessels and lymphoid organs, is an essential regulator of tissue fluid homeostasis, immune cell trafficking, and immunological surveillance [6–8]. Lymphatic vessels can be divided into several subtypes: initial lymphatic with incomplete basement membrane and no pericyte/smooth-muscle-cell coverage; transitional precollecting lymphatics; and larger collecting lymphatics with a complete basement membrane and smooth muscle investment. In the context of cancers, these different types of lymphatic vessels can be actively regulated by tumor-derived growth factors [8,9]. Lymphangiogenesis, the formation of new lymphatic vessels, has been associated with metastasis of solid tumors to lymph nodes (LNs) and distant organs [8–13]. Recent studies demonstrate that lymphatic vessels undergo dynamic remodeling, including lymphangiogenesis and lymphatic enlargement, which facilitates tumor metastasis [14–16]. Furthermore, two recent studies further confirmed the dissemination of cancer cells from LN to distant organs through LN blood vessels in tumor-bearing mice [17,18]. Previous studies using various transgenic mouse models employing vascular endothelial growth factor C/D (VEGF-C/D) overexpression or VEGF-C/D trap suggested a potential role for lymphangiogenesis in cancer progression [19–24]. Given that VEGF-C/D can also target nonlymphatic processes, we aim at establishing new mouse models that can specifically target lymphangiogenesis via genetic depletion of proliferating lymphatic endothelial cells (LECs).

Lymphatic vessel markers include Prox1 [25], the lymphatic vessel endothelial hyaluronan receptor-1 (LYVE1) [26], podoplanin (PDPN), and VEGF receptor 3 (VEGFR3) [27]. PDPN, a 43-kDa membrane protein, is present in podocytes [28] and is one of the most widely employed markers of LECs [29,30]. To functionally evaluate the specific role of lymphatic vessels in cancer progression and metastasis, we generated novel transgenic mice that express the herpes simplex virus (HSV) thymidine kinase (tk) under the control of the *PDPN* gene promoter (PDPN-tk mice). Upon ganciclovir (GCV) administration to PDPN-tk mice, PDPN-positive cells that also express tk will convert GCV into a nucleoside analog that irreversibly arrests DNA replication, resulting over time in the depletion of proliferating PDPN-expressing LECs. Here, we demonstrate that the depletion of proliferating PDPN-expressing LECs significantly inhibits lymphangiogenesis in mammary tumors, resulting in decreased distant metastasis without an impact on primary tumor growth.

## Results

### Generation and characterization of the PDPN-Cre and PDPN-tk mice

**Fig 1:**
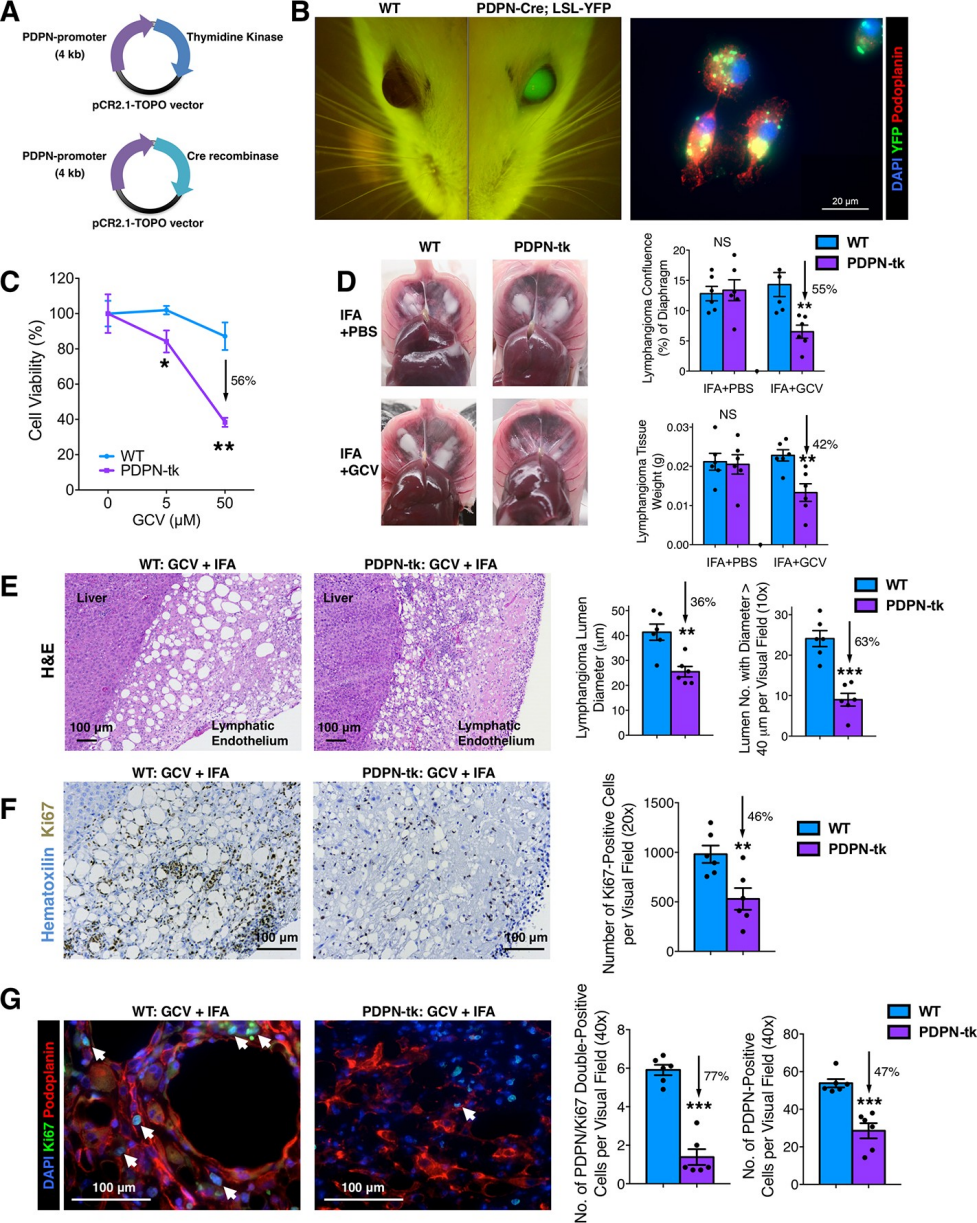
Characterization of PDPN-tk and PDPN-Cre transgenic mice. (A) Schematics demonstrating the generation of PDPN-tk mouse strain or PDPN-Cre mouse strain with a 4-kb *PDPN* promoter sequence ligated to HSV viral tk sequence or Cre recombinase sequence, respectively, using the pCR2.1-TOPO vector. (B) YFP visualization of the eyes of PDPN-Cre; LSL-YFP and control mice. The expression of YFP and PDPN was examined in primary LECs isolated from IFA-induced benign lymphatic endothelial tumor (lymphangioma) in the PDPN-Cre; LSL-YFP mice. Scale bar, 20 μm. (C) Cell viability assay of GCV-treated primary LECs isolated from IFA-induced lymphangioma in PDPN-tk or WT control mice. (D) The comparison of IFA-induced lymphangioma formation in GCV-treated PDPN-tk mice, as compared with WT mice or PDPN-tk mice without GCV treatment (*n* = 6 mice per group). GCV treatment was conducted as daily intraperitoneal injections of 50 mg/kg body weight of GCV. (E) Histology of the lymphangioma tissue samples from PDPN-tk and WT mice. (F) Ki67 staining on the lymphangioma tissue samples from PDPN-tk and WT mice. (G) Immunofluorescence staining of PDPN (red) and Ki67 (green) on the lymphangioma tissues from PDPN-tk and WT mice. White arrows indicate the nuclei of PDPN and Ki67 double positive cells. Scale bars (E–G), 100 μm. Data are represented as mean ± SEM. Significance is determined using an unpaired two-tailed Student *t* test (*p < 0.05, **p < 0.01, ***p < 0.001). The underlying data can be found in S1 Data. Cre, Cre recombinase transgene; GCV, ganciclovir; H&E, hematoxylin and eosin; HSV, herpes simplex virus; IFA, incomplete Freund’s adjuvant; Ki67, cell proliferation antigen Ki-67; LEC, lymphatic endothelial cell; LSL, LoxP-Stop-LoxP; NS, not significant; pCR2.1-TOPO, topoisomerase I-activated pCR2.1-TOPO vector; PDPN, podoplanin; tk, thymidine kinase; WT, wild type; YFP, yellow fluorescent protein.

The PDPN-tk mouse model (BALB/c background) was generated using a 4-kb *PDPN* promoter sequence cloned and ligated to HSV viral tk sequence using the topoisomerase I-activated pCR2.1-TOPO (pCR2.1-TOPO) vector. The PDPN-Cre mouse model was generated using the same *PDPN* promoter sequence cloned and ligated to Cre recombinase sequence (**Fig 1A**). The final constructs were confirmed by DNA sequencing. To examine the specificity of the *PDPN* promoter, we generated the PDPN-Cre; LoxP-Stop-LoxP (LSL)-yellow fluorescent protein (YFP) transgenic mice (BALB/c background) to lineage trace the PDPN^+^ cells. The YFP expression colocalized with LYVE1- or PDPN-positive lymphatic endothelium in normal organs (**S1 Fig**). Additionally, the eyes of PDPN-Cre; LSL-YFP mice exhibited YFP/green fluorescent protein (GFP) expression (**Fig 1B**), consistent with previous observation using Prox1-GFP transgenic mice [31]. Primary LECs were isolated as previously documented [32] from incomplete Freund’s adjuvant (IFA)-induced benign mouse lymphangioma. Briefly, LECs were isolated from hyperplastic lymphatic vessels, cultured, and expanded (**S2A Fig**). These cells exhibited typical LEC morphology, intrinsic YFP expression, and positive immunostaining for PDPN (**Fig 1B**) and LYVE1 (**S2B Fig**). Robust expression of intrinsic YFP was observed in LECs from IFA-induced lymphangioma in PDPN-Cre; LSL-YFP mice, showing the YFP-expressing LECs as the dominant cell population (80% of all nucleated cells) within the lymphangioma tissue (**S2C Fig**). These results confirmed the recombination efficacy of PDPN-Cre in LECs.

Our previous study identified that blood-vascular endothelial-cell–specific deletion of β1 integrin (Tie2-Cre; β1 integrin (Int)^loxP/loxP^ mice) resulted in embryonic lethality due to severe vascular defects [33], while others demonstrated that blood-vascular endothelial-cell–specific deletion of transforming growth factor (TGF) β type II receptor (cadherin 5 promoter-driven tamoxifen-inducible Cre recombinase transgene (Cdh5-CreERT2); TGFBRII^loxP/loxP^ mice) also resulted in embryonic lethality [34]. In contrast, PDPN-Cre; β1 Int^loxP/loxP^ and PDPN-Cre; TGFBRII^loxP/loxP^ mice were born in the expected Mendelian ratio without any noticeable abnormality/defect (**S3A** and **S3B Fig**), supporting the specificity of the PDPN-Cre transgenic in targeting gene deletion in lymphatic vessels and not blood vessels.

To evaluate the efficacy of the PDPN-tk transgene, PDPN^+^ LECs separated from lymphangioma tissues of PDPN-tk or wild-type (WT) mice were cultured (**S4A Fig**) and treated with increasing concentrations of GCV (**Fig 1C**). A dose-dependent depletion of LECs (derived from PDPN-tk mice but not WT mice) was observed, reaching 56% of LEC depletion at an exposure of 50 μM GCV. In addition, in vivo administration of GCV to PDPN-tk mice (daily 50 mg/kg body weight) inhibited the formation of IFA-induced lymphangioma when compared to control (WT) mice (**Fig 1D**). As previously documented [32], IFA-induced benign mouse lymphangioma formed white solid masses on the abdominal surface of the diaphragm and on the surface (under the Glisson’s capsule) of the liver (**Fig 1D**). The hyperplastic LECs forming these masses present with enlarged lumens that are distinct from adipose tissue (**S4B Fig**). LEC lumen formation within lymphangioma tissue was specifically impaired in the PDPN-tk mice when compared to control mice (**Fig 1E**), indicative of the depletion of hyperplastic LECs. A significant and specific decrease in proliferating LECs is recorded in lymphangioma of PDPN-tk mice compared to control mice (**Fig 1F** and **1G**). Notably, lymphangioma formation was not altered in control mice, including WT mice (with or without GCV treatment) and non-GCV-treated PDPN-tk mice. The depletion of proliferating LECs resulted in a decrease in the size of lumen structures of lymphangioma, and this was accompanied with a modest increase in α-smooth muscle actin (αSMA)-expressing myofibroblasts in these benign lesions (**S4C Fig**). However, these myofibroblasts did not appear to play a role in IFA-induced lymphangioma because the formation of these lesions was not impaired in αSMA-tk transgenic mice (depletion of proliferating myofibroblasts that exhibit αSMA expression [35], **S4D Fig**). These results underscore the specificity of PDPN-tk mice and support that LEC proliferation, but not myofibroblast proliferation, is essential for the formation of IFA-induced lymphangioma.

### Lymphatic suppression in PDPN-tk mice with GCV treatment

**Fig 2:**
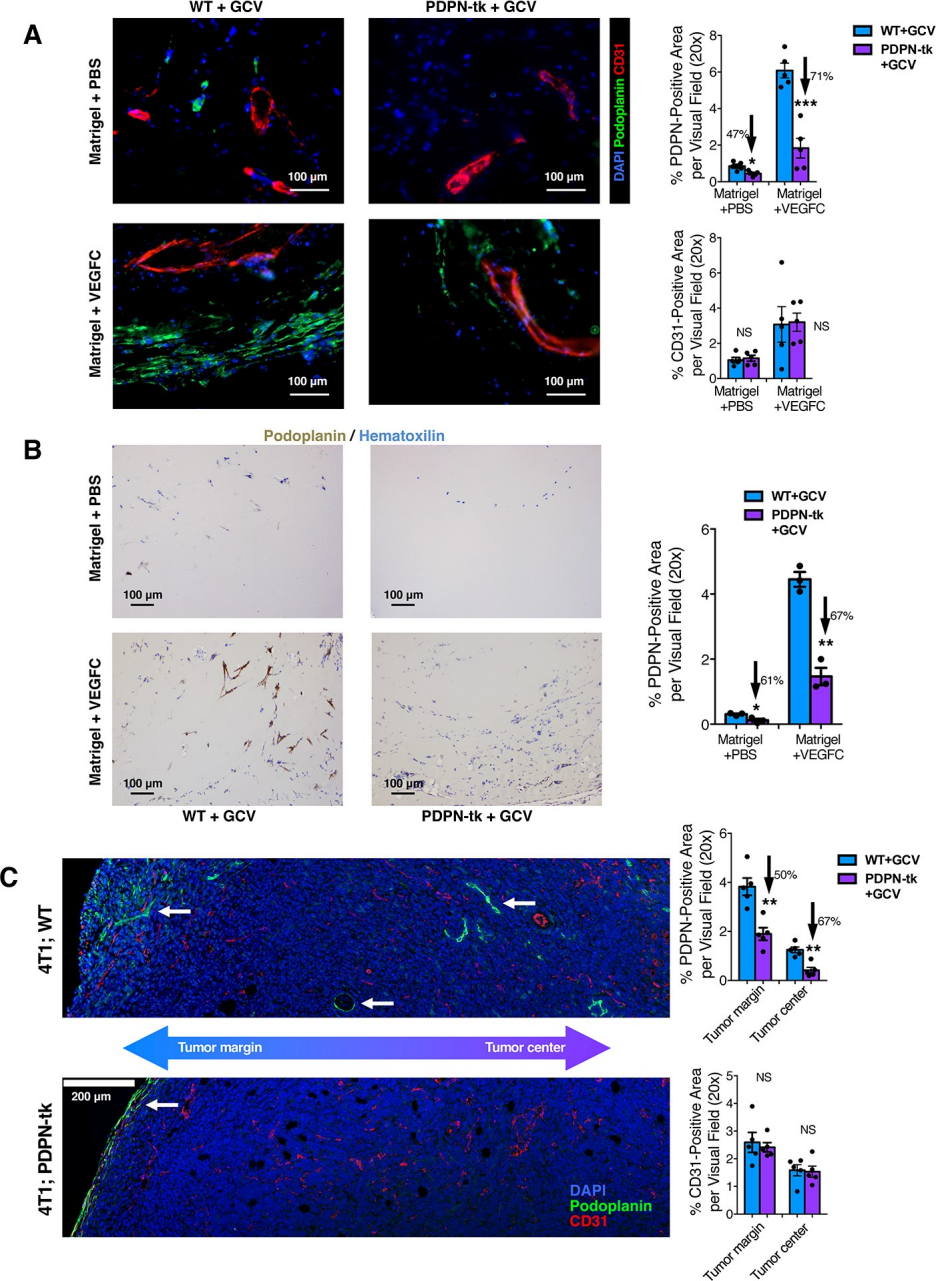
Depletion of proliferating LECs inhibits lymphangiogenesis. (A and B) Lymphangiogenesis and/or angiogenesis in subcutaneously implanted matrigel plugs (growth-factor reduced, supplied with VEGF-C or PBS) from PDPN-tk or WT mice (*n* = 5 mice per group). Lymphatic vessel density and blood vessel density was examined by PDPN and CD31 immunofluorescence staining (A). Overall lymphatic vessels density was also evaluated by IHC staining for PDPN (B). Scale bars (A-B), 100 μm. (C) Orthotopic 4T1 mammary tumors in PDPN-tk or WT mice (*n* = 5 female mice per group) examined for lymphatic vessel density (PDPN staining) and blood vessel density (CD31 staining) in both the margin and intratumoral regions of tumors. Scale bar, 200 μm. GCV treatment was conducted as daily intraperitoneal injections of 50 mg/kg body weight of GCV. The white arrows point to the PDPN-expressing lymphatic vessels. These data are also depicted in S6B Fig. Data are represented as mean ± SEM. Significance is determined using an unpaired two-tailed Student *t* test (*p < 0.05, **p < 0.01, ***p < 0.001). The underlying data can be found in S1 Data. CD, cluster of differentiation; GCV, ganciclovir; IHC, immunohistochemistry; LEC, lymphatic endothelial cell; NS, not significant; PDPN, podoplanin; tk, thymidine kinase; VEGF-C, vascular endothelial growth factor C; WT, wild type

Matrigel plug assay was conducted to determine the functional role of PDPN^+^ LECs in lymphangiogenesis. Growth-factor–reduced matrigel supplemented with VEGF-C induced robust lymphatic vessel formation as well as blood vessel formation after subcutaneous implantation (400 μL matrigel per plug; one plug per mouse). In contrast with WT + GCV mice, PDPN-tk + GCV mice exhibited reduced lymphangiogenesis (**Fig 2A**) and LEC proliferation (**S5A Fig**) in the matrigel plugs, while the angiogenesis response, measured by cluster of differentiation (CD) 31 immunolabeling, was unaffected (**Fig 2A**). Decreased lymphatic vessel density in matrigel plugs of PDPN-tk mice was also confirmed by immunohistochemical assessment of lymphatic markers, PDPN (**Fig 2B)** and LYVE1 (**S5B Fig**). The PDPN-expressing cells within the matrigel plugs were predominantly co-immunolabeled with the LEC marker LYVE1 but did not express the cancer-associated fibroblast marker αSMA (**S5C Fig**). These results support that the cell population targeted by the PDPN-tk transgene in the aforementioned matrigel plug assays comprises of LECs and not fibroblasts.

Additionally, we examined the LN, intestine, and kidney of WT + GCV mice and PDPN-tk + GCV mice bearing the VEGF-C enriched matrigel plug. Although specific depletion of PDPN-expressing LECs was observed in the plug with active lymphangiogenesis (**Fig 2A**), no changes were noted for PDPN immunolabeling in these normal, unaffected tissues (**S6A Fig**), supporting that our genetic strategy only targets proliferating PDPN-expressing cells.

### Depletion of LECs inhibits lung metastasis but not primary mammary tumor growth

**Fig 3:**
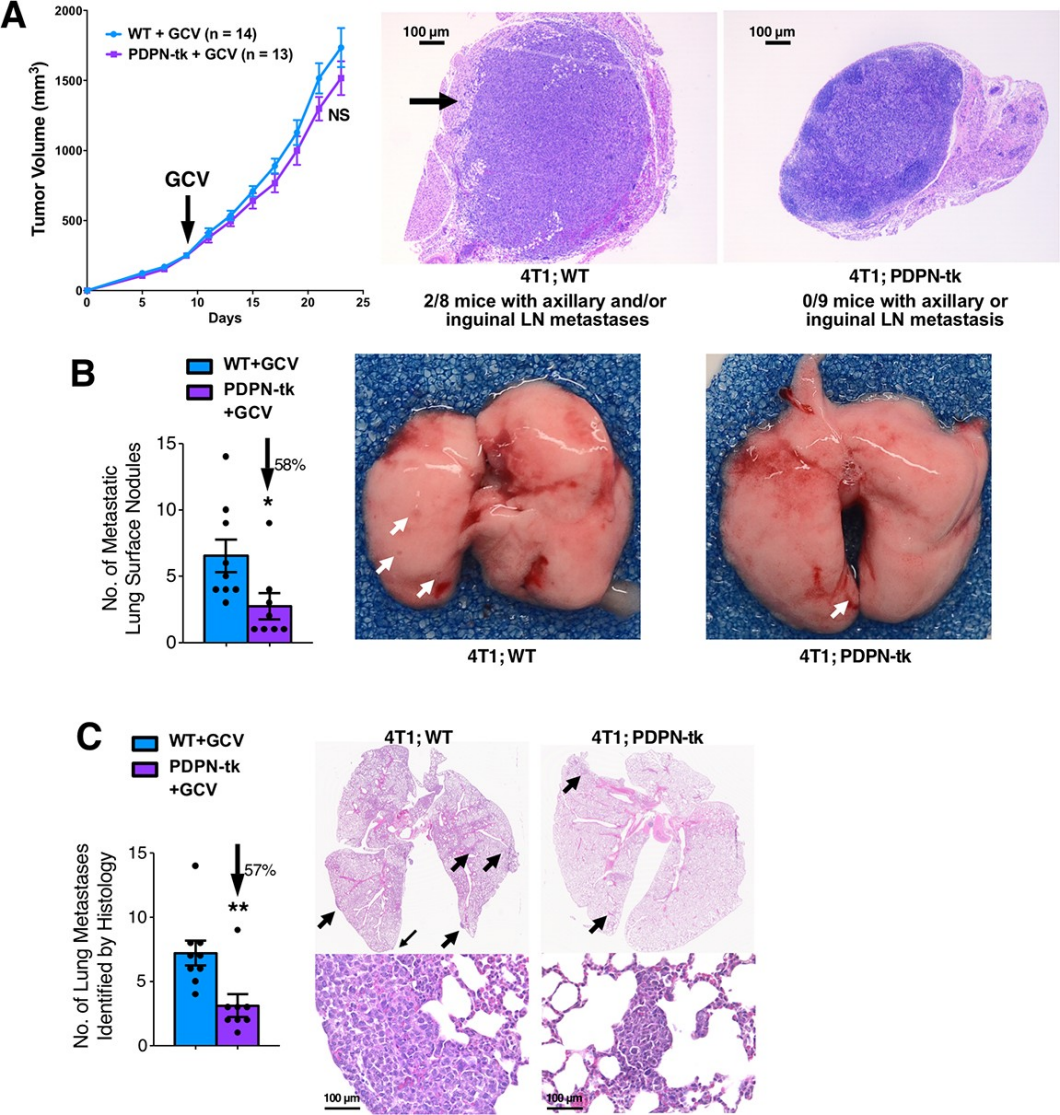
Lung metastasis from 4T1 mammary tumor is inhibited by suppression of lymphangiogenesis. (A) The growth of orthotopic 4T1 mammary tumors in GCV-treated PDPN-tk (*n* = 13) or WT (*n* = 14) female mice. GCV label indicates the starting day of GCV treatment. GCV treatment was conducted as daily intraperitoneal injections of 50 mg/kg of GCV. The identification of tumor-invaded axillary and/or inguinal LNs by histology in 4T1; WT (*n* = 8) mice or 4T1; PDPN-tk (*n* = 9) mice. (B and C) The evaluation of surface metastatic lung nodules (B, white arrows) and histologically identified lung metastases (C, black arrows) in PDPN-tk or WT female mice bearing 4T1. The underlying data can be found in S1 Data. GCV, ganciclovir; LN, lymph node; PDPN, podoplanin; tk, thymidine kinase; WT, wild type

Tumor lymphangiogenesis was examined in orthotopic 4T1 mammary tumors established in either PDPN-tk or WT female mice (all treated with GCV). Tumor tissues were scanned for lymphatic vessels and blood vessels. GCV-treated PDPN-tk mice revealed significantly suppressed lymphangiogenesis in both tumor center and tumor margin/periphery, defined as 100 μm from the tumor edge [36,37], while angiogenesis was not significantly altered (**Fig 2C, S6B Fig,** and **S7A Fig**). Despite a significant suppression of lymphangiogenesis, the growth of orthotopic 4T1 mammary tumors in GCV-treated PDPN-tk mice was unchanged when compared with GCV-treated WT control mice (**Fig 3A**). Interestingly, despite the unchanged primary tumor growth, PDPN-tk-GCV mice with tumors exhibited significantly fewer surface metastatic lung nodules (**Fig 3B**) and histologically identified lung metastases (**Fig 3C**) when compared to WT mice. No tumor-infiltrated axillary or inguinal LN was observed in PDPN-tk-GCV mice (0 out of 9 mice), whereas WT mice in the control group occasionally presented with axillary and/or inguinal LN metastases (2 out of 8 mice) (**Fig 3A**).

**Fig 4:**
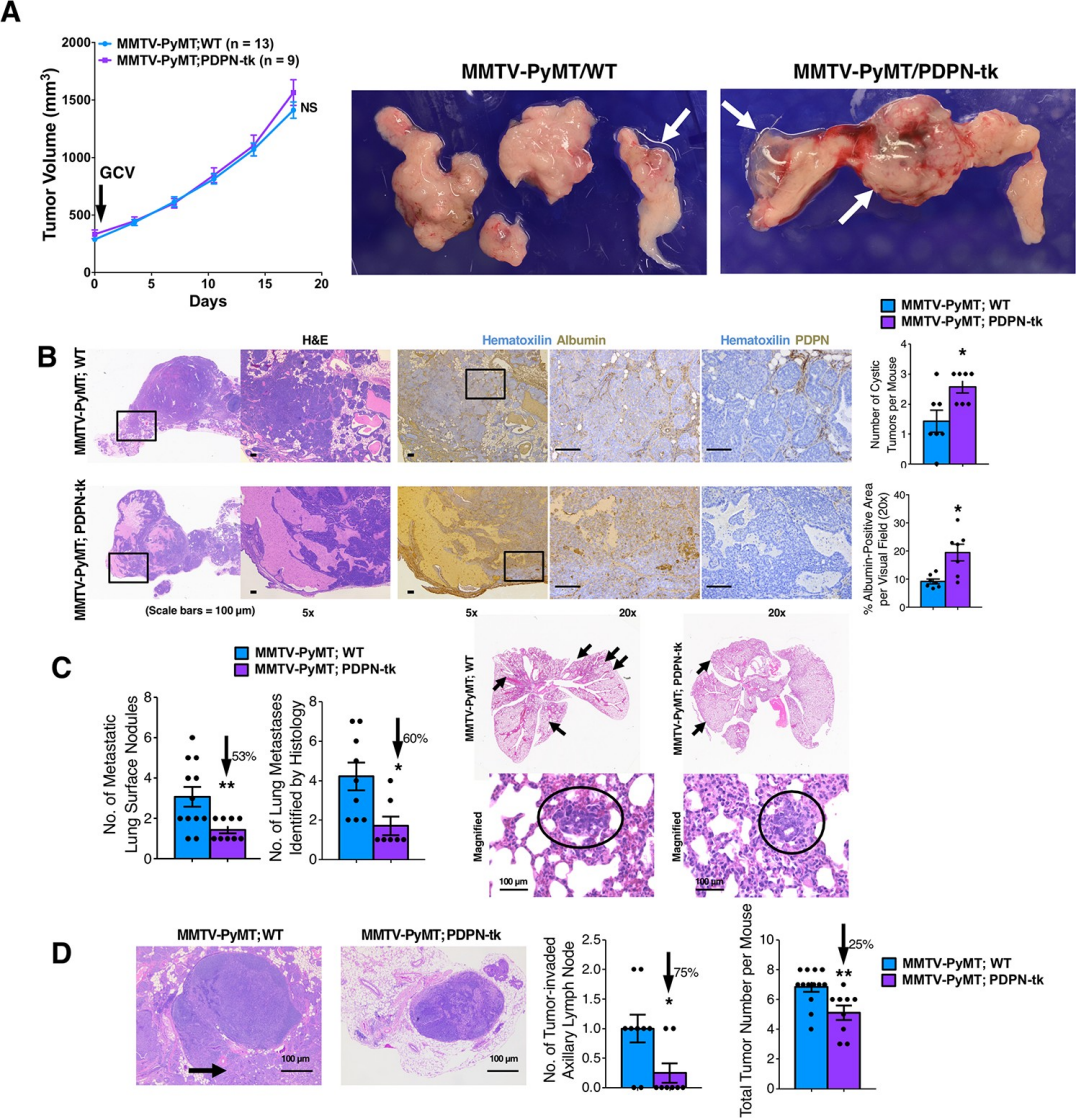
Lung and LN metastasis from MMTV-PyMT mammary tumor is inhibited by suppression of lymphangiogenesis. (A) The growth of spontaneous mammary tumors in GCV-treated MMTV-PyMT; PDPN-tk mice (*n* = 9) or MMTV-PyMT; WT female mice (*n* = 13). GCV label indicates the starting day of GCV treatment. Cystic tumors are indicated by white arrows in the representative pictures of excised tumors. (B) H&E and IHC (albumin or PDPN) stainings of MMTV-PyMT; PDPN-tk or MMTV-PyMT; WT tumors. Number of cystic tumors per mouse were shown for MMTV-PyMT; PDPN-tk or MMTV-PyMT; WT groups. Albumin accumulation was quantified from IHC staining images for both groups. (C) The evaluation of surface metastatic lung nodules and histologically identified lung metastases in MMTV-PyMT; PDPN-tk mice or MMTV-PyMT; WT mice. (D) The number of tumor-invaded axillary LNs and total number of tumors per mouse in MMTV-PyMT; PDPN-tk mice or MMTV-PyMT; WT mice. Scale bars (C-G), 100 μm. Data are represented as mean ± SEM. Significance is determined using an unpaired two-tailed Student *t* test (*p < 0.05, **p < 0.01). The underlying data can be found in S1 Data. GCV, ganciclovir; H&E, hematoxylin and eosin; IHC, immunohistochemistry; LN, lymph node MMTV-PyMT, mouse mammary tumor virus–polyoma middle tumor antigen; NS, not significant; PDPN, podoplanin; tk, thymidine kinase; WT, wild type

We also employed the mouse mammary tumor virus–polyoma middle tumor antigen (MMTV-PyMT) model, in which spontaneous mammary carcinomas and lung metastasis develop, to examine the impact of lymphatic/LEC depletion on cancer progression. MMTV-PyMT mice were bred with PDPN-tk mice to generate the MMTV-PyMT; PDPN-tk mice, as well as the MMTV-PyMT; WT littermate control mice. Female mice were monitored for tumor growth. The growth of MMTV-PyMT tumors was not significantly altered in GCV-treated MMTV-PyMT; PDPN-tk mice when compared to MMTV-PyMT; WT mice (**Fig 4A**). Decreased lymphatic vessel density in MMTV-PyMT; PDPN-tk tumors was confirmed by immunohistochemical staining for LYVE1 (**S7B Fig**). The MMTV-PyMT; PDPN-tk mice exhibited increased incidence of cystic tumors, possibly resulting from enhanced lymphedema, when compared to control mice (**Fig 4A** and **4B**). Previous studies have established that impaired lymphatic function can result in the accumulation of macromolecular proteins (such as albumin) because of compromised lymphatic drainage [38–40]. The increased level of lymphedema in primary tumor tissues of MMTV-PyMT; PDPN-tk mice was confirmed by albumin immunohistochemistry (**Fig 4B**). The total number of surface metastatic lung nodules, histologically identified lung metastatic lesions, and axillary LN metastasis was significantly reduced in MMTV-PyMT; PDPN-tk mice when compared to MMTV-PyMT; WT mice (**Fig 4C and 4D**). Taken together, these results support that suppression of lymphangiogenesis in primary mammary tumors did not impact their growth but limited their metastatic dissemination. Previous studies indicated that PDPN may also be expressed by cancer-associated fibroblasts [41–43] or macrophages [44]. Our analyses revealed that PDPN-expressing cells did not coexpress the breast-tumor–associated fibroblast marker αSMA but predominantly coexpressed the LEC-associated marker VEGFR3 (**S7C Fig**) and weakly coexpressed or failed to coexpress the vascular marker CD31 (**S7D Fig**), consistent with previous observations [19]. We also noted that PDPN^+^ cells did not show colocalization with the macrophage marker CD68 in 4T1 tumors (WT mice), although close contact between CD68^+^ macrophages and PDPN^+^/LYVE1^+^ lymphatic vessels could be occasionally observed (**S7E Fig**).

**Fig 5:**
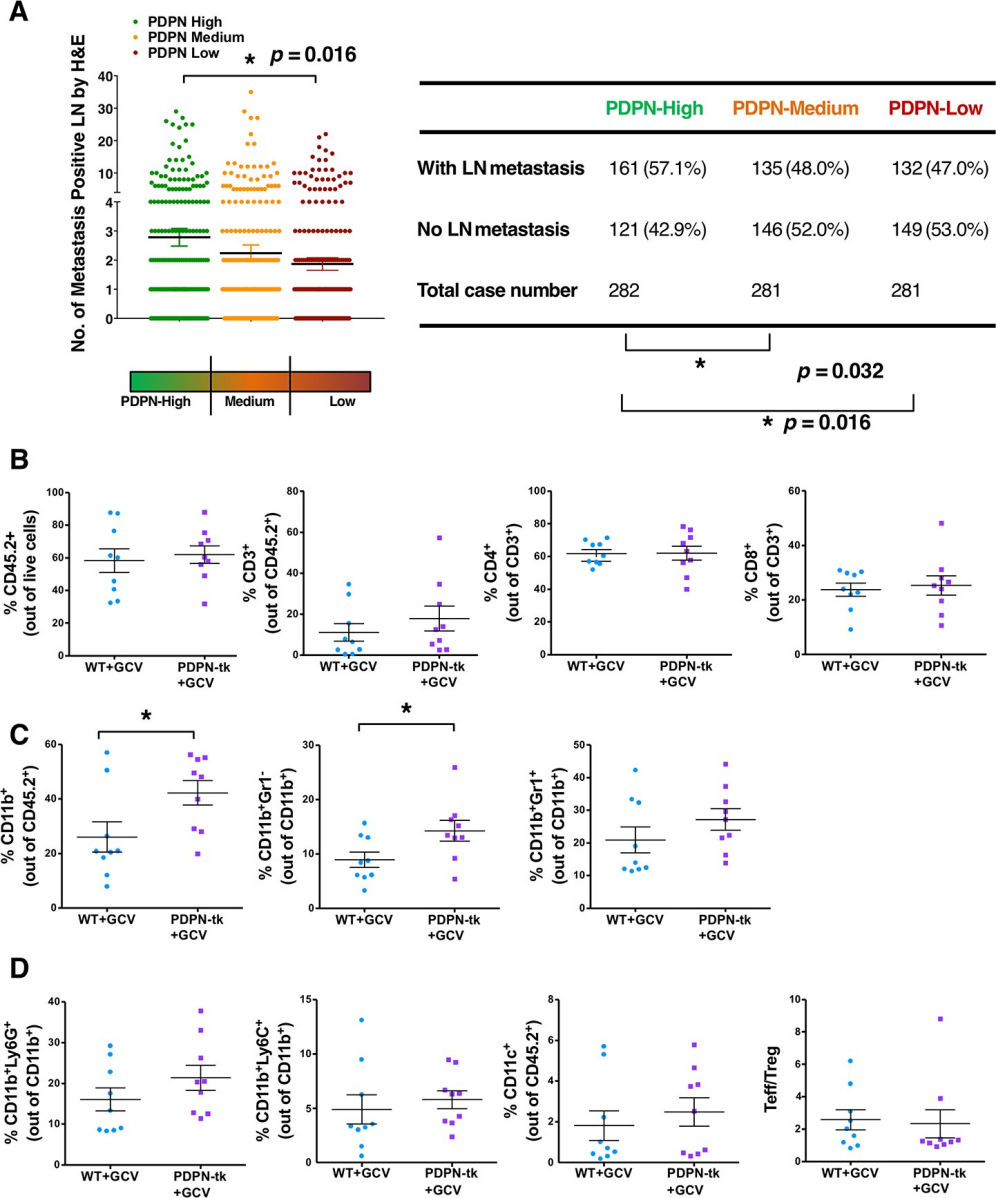
Tumor immune profile when lymphangiogenesis is suppressed. (A) The correlation between PDPN-relative mRNA level and LN metastasis in 844 breast cancer patients with available data for both RNA Seq V2 RSEM and examined LN metastasis count (identified by H&E) from TCGA breast invasive carcinoma data set. PDPN level is presented as relative mRNA expression normalized to the *Gapdh* housekeeping gene. A table presenting the number of LN metastasis positive cases out of total cases is also shown (χ^2^ analysis). (B) Percentages of CD45^+^, CD3^+^, CD4^+^, and CD8^+^ cells in 4T1 orthotopic mammary tumors of PDPN-tk or WT mice (*n* = 9 mice per group). (C) Percentages of CD11b^+^, CD11b^+^ Gr1^−^ (Ly6C^−^Ly6G^−^), and CD11b^+^Gr1^+^ cells in 4T1 orthotopic mammary tumors of PDPN-tk or WT mice (*n* = 9 mice per group). (D) Percentages of CD11b^+^Ly6G^+^, CD11b^+^Ly6C^+^, CD11c^+^ cells, and ratio of CD4^+^FoxP3^−^ Teffs to CD4^+^FoxP3^+^ (Treg) in 4T1 orthotopic mammary tumors of PDPN-tk or WT mice (*n* = 9 mice per group). Data are represented as mean ± SEM. Significance is determined using an unpaired two-tailed Student *t* test (*p < 0.05). The underlying data can be found in S1 Data. CD, cluster of differentiation; GCV, ganciclovir; H&E, hematoxylin and eosin; LN, lymph node; PDPN, podoplanin; RNA Seq V2, RNA Sequencing Version 2 analysis; RSEM, normalized gene expression with RSEM output; TCGA, The Cancer Genome atlas; Teff, effector T cell; tk, thymidine kinase; Treg, regulatory T cell; WT, wild type.

Further, The Cancer Genome Atlas (TCGA) data set of 844 patients with invasive breast carcinoma (RNA sequencing version 2 analysis [RNA Seq V2] normalized gene expression with RSEM output [RSEM]) revealed a correlation between PDPN mRNA level and LN metastasis, showing higher levels of PDPN mRNA (PDPN mRNA expression normalized to *Gapdh*) associated with more LN metastasis (**Fig 5A**). These results were consistent with previous reports regarding the correlation between PDPN level (as examined by immunohistochemistry) and LN metastasis in breast cancer patients [45,46]. We also found marginally decreased occurrence of metastasis in distant organs (such as bone and lung) in PDPN-low patients compared to PDPN-high patients (**S8A Fig**). However, the number of cases with known distant organ metastasis was too low to offer conclusive evidence regarding the correlation between PDPN level and occurrence of distant metastases.

### Depletion of PDPN^+^ lymphatics leads to increased intratumoral macrophages without an impact on B cells and T cells

Given that lymphatic vessels support immune cell trafficking, we next examined the immune infiltration in lymphatic-depleted tumors compared to control tumors. We employed an established flow-cytometry–based analysis (**S8B Fig**), as previously detailed [35,47]. Upon depletion of PDPN-expressing LECs, the frequencies of most immune cell subpopulations (CD45^+^, CD3^+^, CD4^+^, CD8^+^, CD19^+^, and natural killer [NK] 1.1^+^ cells) remained unaltered (**Fig 5B** and **S8C Fig**). In contrast, the percentage of CD11b^+^ and CD11b^+^Gr1^−^ macrophage population significantly increased in lymphatic-depleted tumors compared to control tumors (**Fig 5C**). This result is consistent with the reports that suggest that elevated macrophage accumulation is associated with lymphedema [48,49]. The percentage of CD11b^+^Gr1^+^-myeloid–derived suppressor cells (either CD11b^+^Ly6G^+^ or CD11b^+^Ly6C^+^) or CD11c^+^ dendritic cells remained unchanged (**Fig 5C**). The ratio of CD4^+^FoxP3^+^ effector T cells (Teff) to CD4^+^FoxP3^+^ regulatory T cells (Treg) was not affected (**Fig 5D**).

## Discussion

Lymphangiogenesis, the formation of new lymphatic vessels, is associated with the progression of solid tumors [8–12]. It is known that lymphatics in the tumors are related to distant metastasis and contribute to immune surveillance and tissue fluid homeostasis. In this study, we performed experiments to determine the functional contribution of lymphatic vessels in lung metastasis associated with breast cancer. To achieve this goal, we generated two new transgenic mouse strains that allowed for the selective depletion of proliferating PDPN-positive LECs (PDPN-tk mice) and for the fate mapping/lineage tracing of PDPN-positive LECs (PDPN-Cre mice). The inhibition of lymphangiogenesis employing PDPN-tk mice supports that breast-cancer–associated LN and lung metastasis is in part relying upon dissemination of cancer cells via lymphatic vessels. Interestingly, two recent studies highlighted that the dissemination route of cancer cells from LN to distant organs employs LN blood vessels in tumor-bearing mice [17,18].

In our studies, the vascular density in the mammary tumors was unchanged upon depletion of PDPN^+^ cells. Lineage tracing experiments employing the PDPN-Cre mice showed that PDPN^+^ cells are associated with lymphatic vessels but not the blood vessels. Interestingly, the growth of primary mammary tumors was not markedly altered when lymphangiogenesis was inhibited. These observations are also in alignment with a previous study showing that lymphangiogenesis induced by VEGF-C overexpression facilitates tumor metastasis without contributing to any growth advantage of primary tumor cells [19]. Various cancer types have distinct preferences in metastatic routes (such as a hematogenous route or a lymphatic route), yet the underlying mechanisms of such phenomena are still poorly understood. A recent study demonstrated the hematogenous route for ovarian cancer metastasis [50] in contrast to a peritoneal circulation-facilitated spread as previously proposed. Notably, depletion of lymphatic vessels did not alter the vascular density or lead to suppression of tumor growth but resulted in intratumor lymphedema due to potential imbalance in tissue fluid homeostasis. The new mouse models described herein may prove helpful for future studies related to breast-cancer–associated lymphedema, a substantial clinical problem observed in breast cancer patients.

Although our results support that the newly generated PDPN-tk transgenic mice enable the specific targeting of LECs in various models of lymphangiogenesis, including tumor lymphangiogenesis, it remains possible that immunolabeling for PDPN could be observed in other stromal cells in the tumor microenvironment, including cancer-associated fibroblasts, as noted in human breast cancer tissues [41–43]. The prognostic value of PDPN-expressing mesenchymal cells in the tumor microenvironment remains to be further studied.

The lymphatic system can regulate immune cell trafficking and tissue fluid homeostasis, yet our results indicated that suppression of tumor lymphangiogenesis did not significantly alter tumor immune infiltration. This may reflect a cancer-type–specific observation since it was reported in melanomas of mice lacking dermal lymphatic vessels that lymphatics were critical in establishing tumor-associated inflammation and immunity [24]. The percentage of intratumoral CD11b^+^Gr1^−^ macrophages, however, was significantly elevated with PDPN^+^ LEC depletion. This may reflect a host response to compensate decreased lymphangiogenesis, in particular since macrophages play a role in regulating lymphangiogenesis and releasing lymphangiogenic factors [51–54]. Increased numbers of CD11b^+^Gr1^−^ macrophages in mammary tumors with PDPN^+^ LEC depletion is also consistent with previous reports on increased macrophage infiltration as a hallmark of lymphedema [48,49] and could support a potential role of these cells in metastasis, albeit further study is still needed. Intriguingly, our results suggest that the decreased metastatic burden associated with suppressed lymphangiogenesis may be independent of a lymphocytic polarization in the primary tumor microenvironment.

## Methods

### Ethics statement

Mice were euthanized using CO_2_ inhalation. All mice were maintained under standard housing conditions at the MD Anderson Cancer Center (MDACC) animal facility and the Beth Israel Deaconess Medical Center (BIDMC) animal facility, and all animal procedures were approved by the MDACC Institutional Animal Care and Use Committee and the BIDMC Institutional Animal Care and Use Committee (IACUC number: 1033).

### Mice

The PDPN-tk mouse strain was generated by cloning and ligating the 4-kb *PDPN* promoter sequence to HSV viral tk sequence using the pCR2.1-TOPO vector (Invitrogen, Carlsbad, CA, USA). A similar approach was used to generate the PDPN-Cre mouse strain. Both transgenic mice were generated by the Transgenic Mouse Core Facility at Harvard Medical School. The mice were backcrossed (over 20 generations) and maintained on the BALB/c genetic background. Primers for PDPN-tk genotyping PCR are PDPN-forward 5′-ACCGGAGACATAAATGCCGA-3′ and TK-reverse 5′-AGCACCCGCCAGTAAGTC-3′. Primers for PDPN-Cre genotyping PCR are PDPN-forward 5′-ACCGGAGACATAAATGCCGA-3′ and Cre-reverse 5′-CGCCGCATAACCAGTGAAAC-3′. αSMA-tk mice were generated and characterized in our previous study [55]. TGFBRII flox mice were kindly provided by H. Moses, Vanderbilt University [56]. β1 integrin flox mice were purchased from the Jackson Laboratory (Bar Harbor, ME, USA). Investigators were not blinded to group allocation but were blinded for the histological assessment of phenotypic outcome. No randomization method was used, and no animal was excluded from the analysis. The experimental endpoint is defined as when tumor burden reaches 1,500 mm^3^ or 1.5 cm in diameter (whichever comes first). For the evaluation of surface lung nodules in mouse mammary tumor models, all surfaces of all of the lobes were ascertained for the presence of surface lung nodules. For the microscopic evaluation of lung metastases in mouse mammary tumor models, we counted the number of nodules observed on a single H&E-stained cross section of the lungs.

### Matrigel plug assay

Matrigel plug assay was conducted to determine the functional role of PDPN^+^ LECs in lymphangiogenesis. Growth-factor–reduced matrigel (Corning, Corning, NY, USA) supplemented with VEGF-C (1 μg /400 μL matrigel) was subcutaneously implanted in WT and PDPN-tk mice (400 μL matrigel per plug; one plug per mouse; *n* = 5 mice per group).

### Immunohistochemistry and immunofluorescence

Primary antibodies are as follows: albumin (A90-134A, Bethyl, 1:100), αSMA (M0851, Dako, 1:100), CD31 (ab28364, Abcam, 1:300), CD68 (M0814, Dako, 1:200), Ki67 (RM-9106, Thermo Scientific, 1:400), LYVE1 (ab14917, Abcam, 1:200), PDPN (ab11936, Abcam, 1:400), VEGFR3 (RM0003-5F63, Novus Biologicals, 1:100), and YFP/GFP (ab13970, Abcam, 1:200). For all immunohistochemical stainings, sections were incubated with biotinylated secondary antibody and then streptavidin-HRP (Vector Labs, Burlingame, CA, USA). Counterstaining with hematoxylin was conducted, and DAB positivity was examined in randomly selected visual fields. For all immunofluorescence stainings, sections were incubated with fluorescent-labeled secondary antibodies according to the primary antibody usage. For the YFP staining of tissue samples from PDPN-Cre; LSL-YFP mice, optimized protocols for tissue collection and immunofluorescence staining were used in order to minimize the autofluorescence in the skin and intestine sections. These optimized protocols include: conducting PBS perfusion before collecting the organs from mice; using Sudan Black B (Sigma-Aldrich, St. Louis, MO, USA) incubation on the sections before the staining [57]; blocking with 4% cold water fish gel before primary antibody incubation; and decreased secondary antibody concentration. Staining for αSMA was performed with Mouse-on-Mouse (MOM) kit (Vector Laboratories) following the manufacturer's instructions. The images of at least 3 random visual fields for each sample section were quantified for positive area using NIH ImageJ analysis software (albumin, CD31, Ki67, LYVE1, or PDPN). Quantified values for multiple visual fields were averaged to produce a single value for each animal, which was then averaged again to represent the mean bar for the group in each graph.

### Mouse lymphatic tissue induced by IFA

Either WT or PDPN-tk mice (3 months old, female) were intraperitoneally injected twice (day 1 and day 14) with IFA (200 μL, 1:1 mixed with PBS) to induce the formation of mouse hyperplastic lymphatic tissue (lymphangioma), as previously described [32]. The lymphangioma confluence was quantified as the percentage coverage by lymphangioma area among the total area of the diaphragm, as quantified by ImageJ software. The average diameter of lumen structures within lymphangioma tissues was calculated by measuring 10–20 randomly selected lumens within microscopic (40×) images of H&E-stained tissue slides using ImageJ software. Mouse lymphangioma tissue, formed by hyperplastic lymphatic vessels on the diaphragm and liver of mice in response to IFA treatment, was collected on day 21 and digested with 1 mg/mL collagenase solution (collagenase I:collagenase II = 1:1) at 37°C for 30 min. Cell suspension was filtered and purified for LECs using anti-PDPN antibody (Abcam, ab11936) and Magnetic Dynabeads (Thermo Fisher Scientific, Waltham, MA, USA). Isolated primary mouse LECs were cultured in endothelial cell growth medium (Lonza, Basel, Switzerland). For the detection of YFP-positive LECs in IFA-induced lymphangioma by flow cytometry, mouse lymphangioma tissue was collected, prepared as a single-cell suspension according to the same protocol above, and examined for YFP fluorescence signal by flow cytometry.

### Cell viability assay

For in vitro treatment of GCV, LECs from WT or PDPN-tk mice were isolated using the same method listed above, cultured, treated with 0, 5, or 50 μM GCV for 48 h, and then examined for cell viability (measured as the absorbance at 450 nm by a microplate reader) using the Cell Counting Kit-8 (Dojindo Molecular Technologies, Kumamoto, Japan). Results of cell viability were expressed as percentage of viable cell counts using the control vehicle-treated group as the reference.

### 4T1 orthotopic mammary tumor model

Either WT or PDPN-tk female mice, around 3 months old, were used for orthotopic implantation of 4T1 mammary epithelial cancer cells. 4T1 Cells were from American Type Culture Collection (ATCC) and cultured in DMEM with 10% FBS and 100 U/mL penicillin–streptomycin. Cells were examined monthly to ensure a negative result for mycoplasma test. Mice were anesthetized with ketamine/xylazine, the skin near the mammary gland was incised, and 4T1 cancer cells were injected into the mammary glands (in total, 1 × 10^6^ cells per mouse; 5 × 10^5^ cells at each side, for both left and right sides), as previously described [4]. PDPN-tk and WT control mice were treated with daily intraperitoneal injections of 50 mg/kg body weight of GCV (InvivoGen, San Diego, CA, USA), when the sum of the tumor volumes per mouse reached approximately 300 mm^3^ (approximately 8–9 days post cancer cell inoculation). Tumor volumes were measured every other day using digital calipers and calculated using the equation length × width^2^ × 0.52. Mice were sacrificed when the sum of the tumor volumes reached approximately 1,500 mm^3^ (approximately 22–25 days post-cancer cell inoculation).

### MMTV-PyMT spontaneous mammary tumor model

MMTV-PyMT transgenic mice from the BALB/c genetic background were provided by Dr. Jack Lawler (BIDMC and Harvard Medical School, Boston, MA, USA). MMTV-PyMT mice were bred with PDPN-tk mice to generate the MMTV-PyMT; PDPN-tk mice. Female MMTV-PyMT; PDPN-tk mice and female MMTV-PyMT; WT littermate control mice were used for mammary tumor studies. GCV treatment was conducted as daily intraperitoneal injections of 50 mg/kg body weight of GCV (InvivoGen, San Diego, CA), starting when the sum tumor volumes per mouse reached approximately 300 mm^3^. Tumor volumes were measured twice per week using digital calipers and calculated using the equation length × width^2^ × 0.52. Mice were sacrificed when tumor volume reached approximately 1,500 mm^3^ or 1.5 cm in diameter (whichever came first). A tumor was counted as a cystic tumor when it formed prominent fluid-filled cyst with a volume greater than 100 mm^3^. Tumors and other organs, including the lungs, were collected as previously described [58].

### Flow-cytometry–based immunotyping analysis

For the characterization of immune infiltration, tumors (from 3-month-old WT or PDPN-tk mice harboring 4T1 orthotopic mammary tumors and treated with GCV) were examined by flow-cytometry–based immunotyping methodology (BD LSRFortessa X-20 Cytometer; BD Biosciences, San Jose, CA, USA). Tumors were weighed, minced with gentleMACS Dissociator (Miltenyi Biotec, Bergisch Gladbach, Germany), and digested in 2 mL solution containing 1 mg/mL Liberase TL (Roche, Indianapolis, IN, USA) and 0.2 mg/mL DNase I in RPMI media at 37°C for 30 min. The tissue lysates were filtered through a 100-μm mesh before immunostaining [35,47]. The subsequent single-cell suspension was stained with fixable viability dye eFluor 780, anti-CD45.2 Pacific Blue, anti-CD3 PE-Cy7, anti-CD3 Alexa Fluor 700, anti-FoxP3 Alexa Fluor 700, anti-CD11c eFluor 615, and anti-NK1.1 PE (eBioscience, San Diego, CA, USA); anti-CD4 Qdot 605 (Life Technologies, Gaithersburg, MD, USA); anti-CD8 Brilliant Violet 650, anti-CD11b Brilliant Violet 570, anti-CD19 Qdot655, and anti-F4/80 FITC (BioLegend, San Diego, CA, USA); and anti-Ly6C APC and anti-Ly6G PE-Cy7 (BD Biosciences). The percentage positive cells were analyzed by FlowJo 10.1. Unstained, live/dead stain only, and single-stained beads (eBioscience) served as compensation controls. Singlets were gated using forward-scatter (FSC) height (FSC-H) and FSC area (FSC-A) event characteristics. Data were derived from multiple experiments with 9 mice per group.

### TCGA data set analysis

The mRNA data (RNA Seq V2 RSEM) and clinical data of 844 patients with invasive breast carcinoma from the TCGA data set were obtained using the cBioPortal for Cancer Genomics (http://www.cbioportal.org/) [59]. χ^2^ analyses, using SPSS statistical software, were performed comparing LN metastatic frequency between PDPN-High and PDPN-Low groups of patients. The metastasis occurrence at distant organs of these patients was also analyzed based on the detailed clinical information from TCGA data set.

### Statistics

Statistical analyses of flow cytometry and immunostaining quantifications were performed with unpaired, two-tailed *t* test, one-way ANOVA with Tukey’s multiple comparison test, or Fisher’s exact test with GraphPad Prism (GraphPad Software, San Diego, CA, USA). A *p* value < 0.05 was considered statistically significant. Error bars represent SEM when multiple visual fields were averaged to produce a single value for each animal, which was then averaged again to represent the mean bar for the group in each graph.

## Supporting information

S1 Fig YFP expression driven by PDPN-Cre in PDPN-Cre; LSL-YFP mice. Images showing the LYVE1 or PDPN immunofluorescence staining (red) together with YFP staining (green) in skin and intestine of PDPN-Cre; LSL-YFP mice or Cre-negative; LSL-YFP control mice. Arrows indicate the colocalization between YFP and immunostaining of lymphatics (PDPN or LYVE1). Scale bars, 25 μm. Cre, Cre recombinase transgene; LSL, LoxP-Stop-LoxP; LYVE1, lymphatic vessel endothelial hyaluronan receptor-1; PDPN, podoplanin; YFP, yellow fluorescent protein.
(TIF)

S2 Fig Isolation of mouse LECs from mouse lymphangioma induced by IFA. (A) Schematic showing the procedure of inducing mouse lymphangioma by IFA intraperitoneal injection. (B) The expression of YFP and LYVE1 in primary LECs isolated from IFA-induced lymphangioma in the PDPN-Cre; LSL-YFP mice. Scale bar, 20 μm. (C) IFA-induced mouse lymphangioma was isolated from PDPN-Cre; LSL-YFP mice and examined for YFP-expressing LECs by flow cytometry. In P1: RBCs. In P2: YFP^−^ (non-RBC) cells. In P3: YFP^+^ (non-RBC) cells. Cre, Cre recombinase transgene; IFA, incomplete Freund’s adjuvant; LEC, lymphatic endothelial cell; LSL, LoxP-Stop-LoxP; LYVE1, lymphatic vessel endothelial hyaluronan receptor-1; PDPN, podoplanin; RBC, red blood cell; YFP, yellow fluorescent protein.
(TIF)

S3 Fig The generation of PDPN-Cre; β1 Int^loxP/loxP^ and PDPN-Cre; TGFBRII^loxP/loxP^ mice. (A) Breeding scheme and outcome of crossing PDPN-Cre; β1 Int^loxP/+^ and β1 Int^loxP/loxP^ mice to generate PDPN-Cre; β1 Int^loxP/loxP^ mice. (B) Breeding scheme and outcome of crossing PDPN-Cre; TGFBRII^loxP/+^ and TGFBRII^loxP/loxP^ mice to generate PDPN-Cre; TGFBRII^loxP/loxP^ mice. Cre, Cre recombinase transgene; Int, integrin; PDPN, podoplanin; TGFBRII, transforming growth factor beta receptor II.
(TIF)

S4 Fig Further characterization of mouse lymphangioma induced by IFA. (A) Schematic showing the procedure for the isolation and establishment of mouse primary LEC culture from IFA-induced lymphangioma. (B) The representative images comparing the histology of IFA-induced lymphangioma tissue at the surface of liver and the adipose tissue adjacent to mammary tumor (MMTV-PyMT). Scale bars, 100 μm. (C) Immunofluorescence staining of PDPN (red) and αSMA (white) on the IFA-induced lymphangioma tissues from PDPN-tk and WT mice. Scale bars, 100 μm. (D) The IFA-induced lymphangioma formation was not affected in GCV-treated αSMA-tk mice as compared with GCV-treated WT mice (*n* = 5 mice per group). Data are represented as mean ± SEM. Significance is determined using an unpaired two-tailed Student *t* test. The underlying data can be found in S1 Data. αSMA, α-smooth muscle actin; GCV, ganciclovir; IFA, incomplete Freund’s adjuvant; LEC, lymphatic endothelial cell; MMTV-PyMT, mouse mammary tumor virus–polyoma middle tumor antigen; NS, not significant; PDPN, podoplanin; tk, thymidine kinase; WT, wild type.
(TIF)

S5 Fig Lymphangiogenesis and/or angiogenesis in subcutaneous matrigel plugs. (A–C) Lymphangiogenesis and/or angiogenesis in subcutaneously implanted matrigel plugs (growth-factor reduced, supplied with VEGF-C or PBS) from PDPN-tk or WT mice (*n* = 5 mice per group). The proliferation status of LECs was examined by Ki67 and PDPN immunofluorescence staining (A). Overall lymphatic vessel density was also evaluated by IHC staining for LYVE1 (B). Scale bars (A–B), 100 μm. The cell populations within the matrigel plugs were further examined by PDPN, VEGFR3, and αSMA immunofluorescence staining (C). Scale bars (C), 25 μm. Data are represented as mean ± SEM. Significance is determined using an unpaired two-tailed Student *t* test (*p < 0.05, ***p < 0.001). The underlying data can be found in S1 Data. αSMA, α-smooth muscle actin; IHC, immunohistochemistry; Ki67, cell proliferation antigen Ki-67; LEC, lymphatic endothelial cell; LYVE1, lymphatic vessel endothelial hyaluronan receptor-1; PDPN, podoplanin; tk, thymidine kinase; VEGF-C, vascular endothelial growth factor C; VEGFR3, vascular endothelial growth factor receptor 3; WT, wild type.
(TIF)

S6 Fig Lymphangiogenesis and/or angiogenesis in multiple tissue types of WT and PDPN-tk mice. (A) Images showing the PDPN immunofluorescence staining (red) in the kidney, intestine, and LN of GCV-treated WT and PDPN-tk mice. Scale bars, 100 μm. (B) Orthotopic 4T1 mammary tumors in PDPN-tk or WT mice (*n* = 5 female mice per group) examined for lymphatic vessel density (PDPN staining) and blood vessel density (CD31 staining). These data are also shown in Fig 2C. Scale bar, 100 μm. CD, cluster of differentiation; GCV, ganciclovir; LN, lymph node; PDPN, podoplanin; tk, thymidine kinase; WT, wild type.
(TIF)

S7 Fig Further characterization of lymphangiogenesis in mouse mammary tumors from WT and PDPN-tk mice. (A and B) Representative images of LYVE1 immunohistochemical staining in 4T1 orthotopic mammary tumors (A) or MMTV-PyMT spontaneous mammary tumors (B) from PDPN-tk or WT female mice (*n* = 5 per group). Scale bars, 100 μm. Data are represented as mean ± SEM. Significance is determined using an unpaired two-tailed Student *t* test (*p < 0.05). (C) Representative images of 4T1 and MMTV-PyMT mammary tumors (WT mice) stained for PDPN, VEGFR3, and αSMA. Scale bars, 25 μm. (D) Representative images of 4T1 mammary tumors (WT mice) stained for PDPN, CD31, and αSMA. Scale bars, 25 μm. (E) Representative images of 4T1 mammary tumors stained for PDPN, LYVE1, and CD68. Scale bars, 25 μm. The underlying data can be found in S1 Data. αSMA, α-smooth muscle actin; CD, cluster of differentiation; LYVE1, lymphatic vessel endothelial hyaluronan receptor-1; MMTV-PyMT, mouse mammary tumor virus–polyoma middle tumor antigen; PDPN, podoplanin; tk, thymidine kinase; VEGFR3, vascular endothelial growth factor receptor 3; WT, wild type.
(TIF)

S8 Fig Additional data for the TCGA data set analysis and immune infiltration profile. (A) The correlation between PDPN relative mRNA level and distant organ metastasis in 844 breast cancer patients with available data from TCGA breast invasive carcinoma data set. (B) Representative flow cytometry gating strategies used to analyze mammary tumor immune infiltration profile for T cell panel and myeloid cell panel, respectively. (C) Percentages of CD19^+^ and NK1.1^+^ cells in 4T1 orthotopic mammary tumors of PDPN-tk or WT mice (*n* = 9 per group). The underlying data can be found in S1 Data. CD, cluster of differentiation; NK, natural killer; PDPN, podoplanin; TCGA, The Cancer Genome Atlas; WT, wild type.
(TIF)

S1 Data Source data used for quantification in this study. (XLSX)

## Abbreviations

ATCC: American Type Culture Collection
BIDMC: Beth Israel Deaconess Medical Center
CD: cluster of differentiation
Cdh5-CreERT2: cadherin 5 promoter-driven tamoxifen-inducible Cre recombinase transgene
Cre: Cre recombinase transgene
FSC: forward scatter
FSC-A: FSC-area
FSC-H: FSC-height
GCV: ganciclovir
GFP: green fluorescent protein
HSV: herpes simplex virus
H&E: hematoxylin and eosin
IACUC: Institutional Animal Care and Use Committee
IFA: incomplete Freund’s adjuvant
IHC: immunohistochemistry
Int: integrin
Ki67: cell proliferation antigen Ki-67
LEC: lymphatic endothelial cell
LN: lymph node
LSL: LoxP-Stop-LoxP LYVE1, lymphatic vessel endothelial hyaluronan receptor-1
MDACC: MD Anderson Cancer Center
MMTV-PyMT: mouse mammary tumor virus–polyoma middle tumor antigen
MOM: Mouse-on-Mouse
NK: natural killer
NS: not significant
pCR2.1-TOPO: topoisomerase I-activated pCR2.1-TOPO vector
PDPN: podoplanin
PDPN-Cre: podoplanin promoter-driven Cre recombinase transgene
PDPN-tk: podoplanin promoter-driven thymidine kinase transgene
RNA Seq V2: RNA Sequencing Version 2 analysis
RSEM: normalized gene expression with RSEM output
TCGA: The Cancer Genome Atlas
Teff: effector T cell
TGFBRII: transforming growth factor beta receptor II
TGFβ: transforming growth factor beta
tk: thymidine kinase
Treg: regulatory T cell
VEGF-C/D: vascular endothelial growth factor C/D
VEGFR3: vascular endothelial growth factor receptor 3
WT: wild type
YFP: yellow fluorescent protein
αSMA: α-smooth muscle actin
