# Supplementary figures and images for: Podoplanin+ tumor lymphatics are rate limiting for breast cancer metastasis
Source: PLoS Biol. 2018 Dec 28;16(12):e2005907. doi: 10.1371/journal.pbio.2005907 (PMC6310240; doi:10.1371/journal.pbio.2005907)

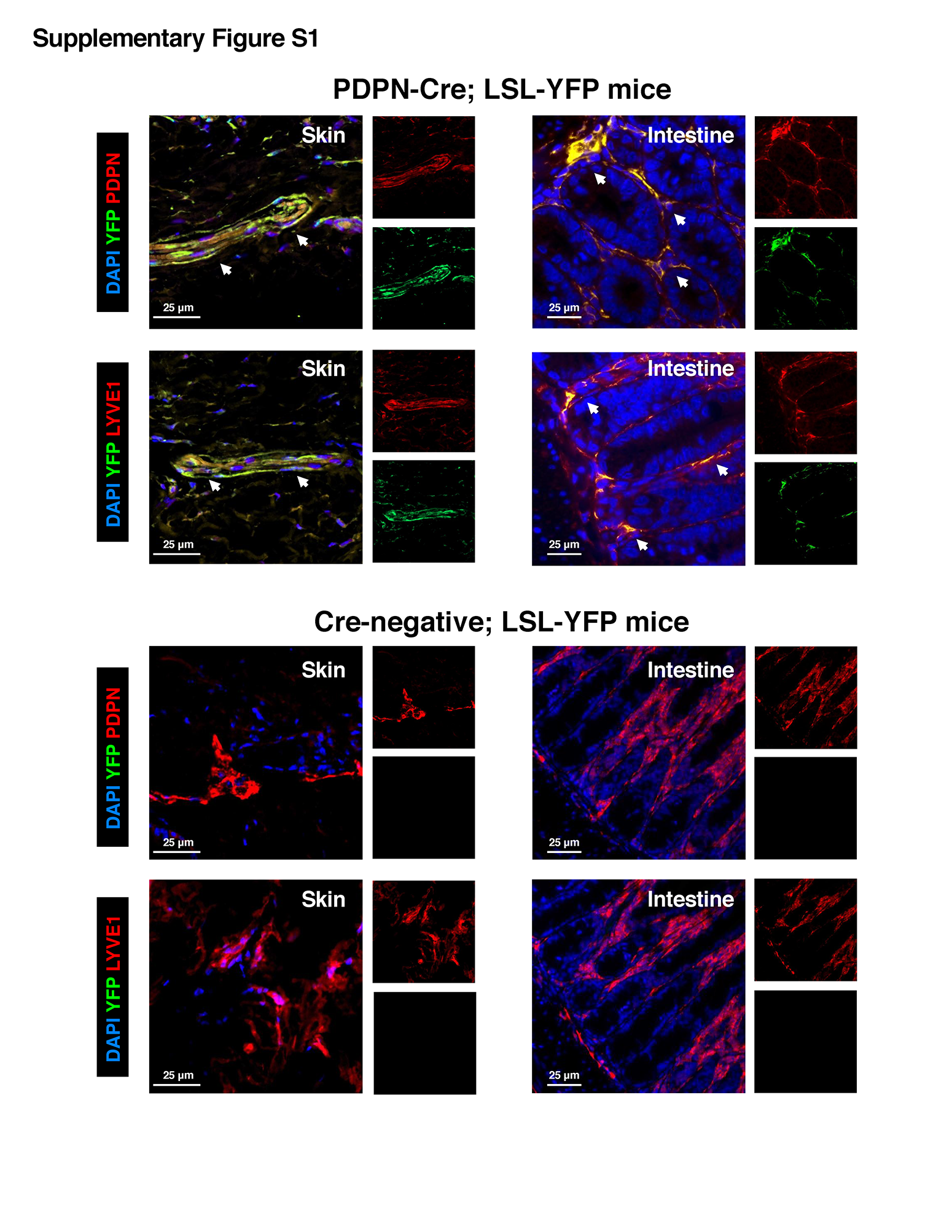

Supplement: S1 Fig — Images showing the LYVE1 or PDPN immunofluorescence staining (red) together with YFP staining (green) in skin and intestine of PDPN-Cre; LSL-YFP mice or Cre-negative; LSL-YFP control mice. Arrows indicate the colocalization between YFP and immunostaining of lymphatics (PDPN or LYVE1). Scale bars, 25 μm. Cre, Cre recombinase transgene; LSL, LoxP-Stop-LoxP; LYVE1, lymphatic vessel endothelial hyaluronan receptor-1; PDPN, podoplanin; YFP, yellow fluorescent protein. (TIF) [file pbio.2005907.s001.tif]

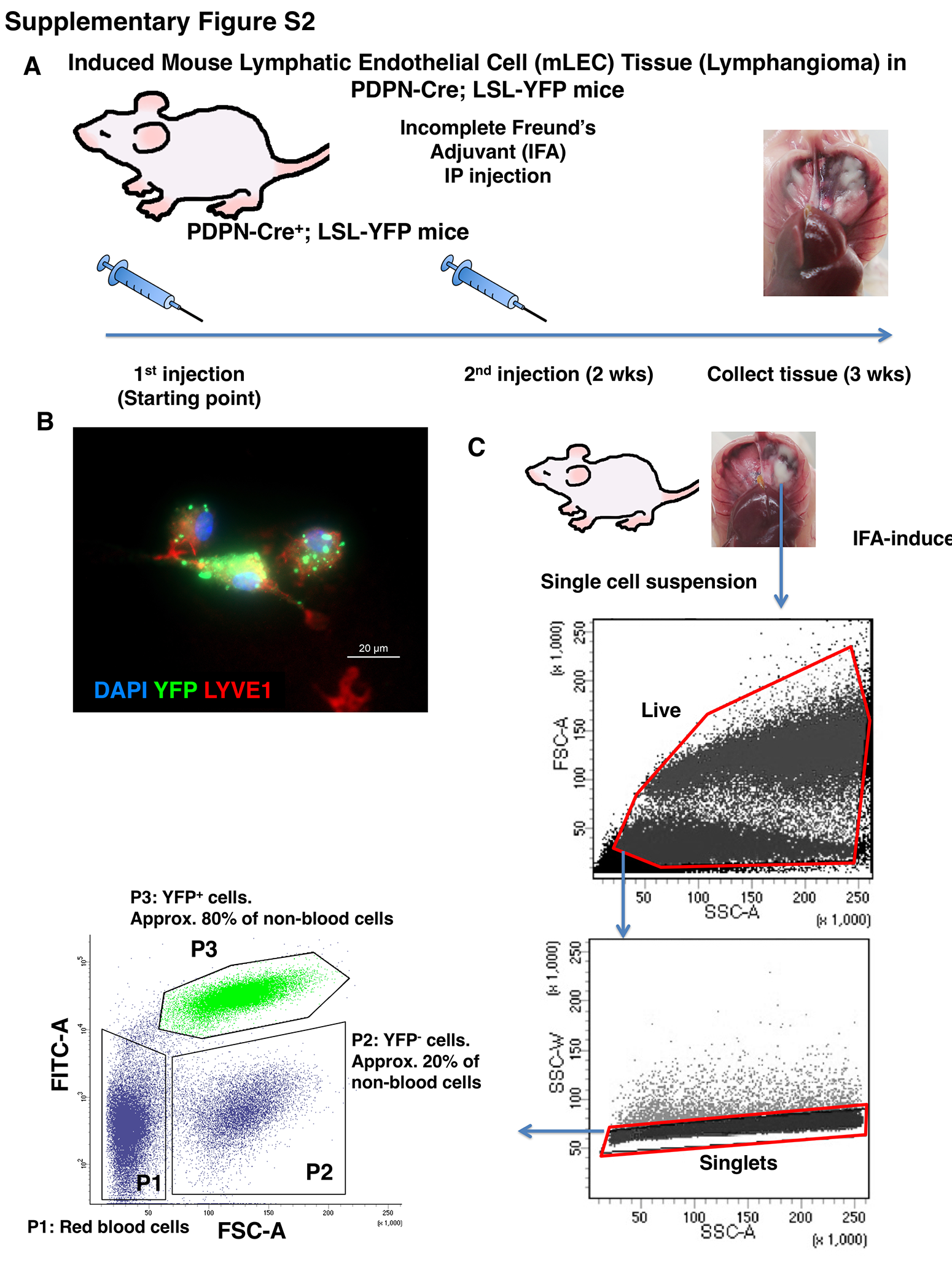

Supplement: S2 Fig — (A) Schematic showing the procedure of inducing mouse lymphangioma by IFA intraperitoneal injection. (B) The expression of YFP and LYVE1 in primary LECs isolated from IFA-induced lymphangioma in the PDPN-Cre; LSL-YFP mice. Scale bar, 20 μm. (C) IFA-induced mouse lymphangioma was isolated from PDPN-Cre; LSL-YFP mice and examined for YFP-expressing LECs by flow cytometry. In P1: RBCs. In P2: YFP− (non-RBC) cells. In P3: YFP+ (non-RBC) cells. Cre, Cre recombinase transgene; IFA, incomplete Freund’s adjuvant; LEC, lymphatic endothelial cell; LSL, LoxP-Stop-LoxP; LYVE1, lymphatic vessel endothelial hyaluronan receptor-1; PDPN, podoplanin; RBC, red blood cell; YFP, yellow fluorescent protein. (TIF) [file pbio.2005907.s002.tif]

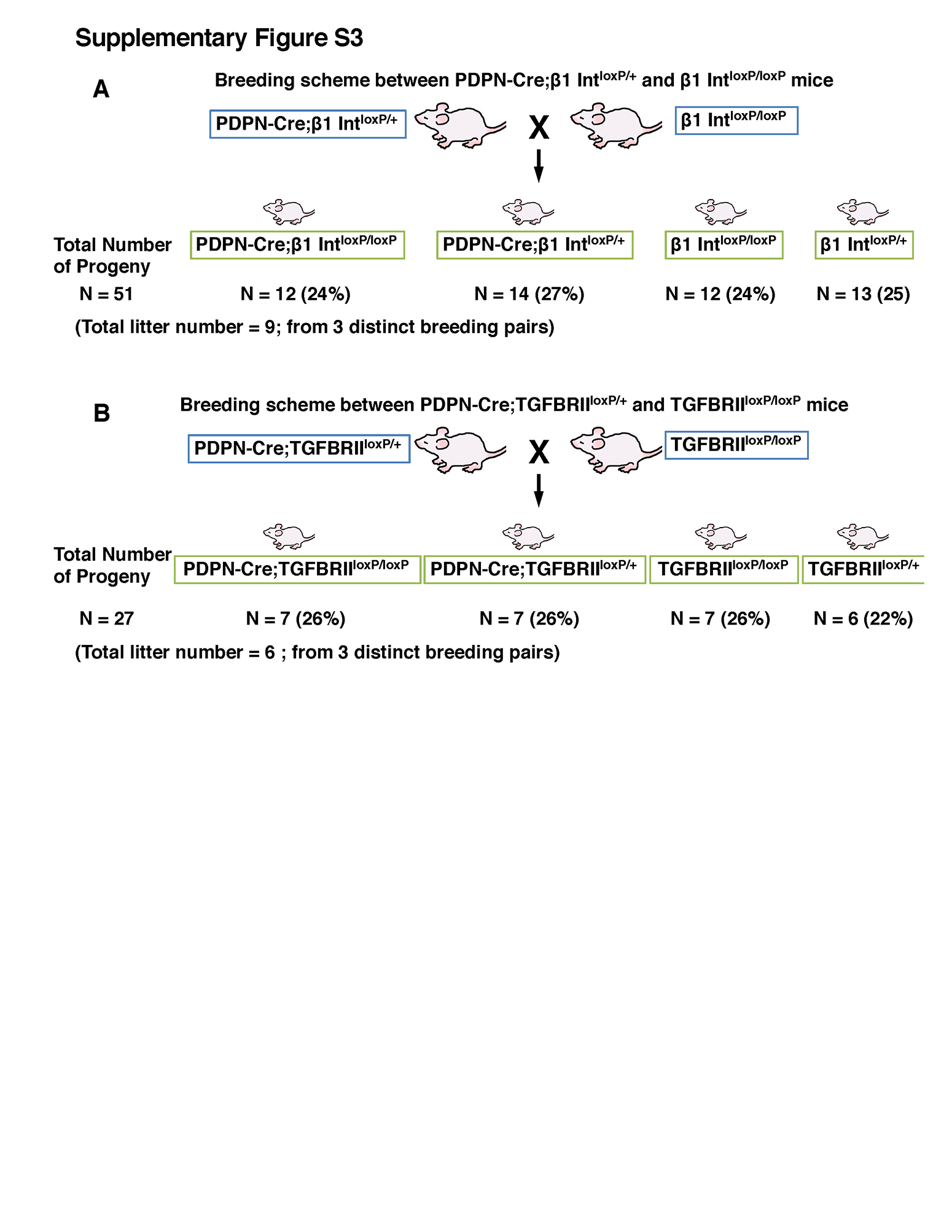

Supplement: S3 Fig — (A) Breeding scheme and outcome of crossing PDPN-Cre; β1 IntloxP/+ and β1 IntloxP/loxP mice to generate PDPN-Cre; β1 IntloxP/loxP mice. (B) Breeding scheme and outcome of crossing PDPN-Cre; TGFBRIIloxP/+ and TGFBRIIloxP/loxP mice to generate PDPN-Cre; TGFBRIIloxP/loxP mice. Cre, Cre recombinase transgene; Int, integrin; PDPN, podoplanin; TGFBRII, transforming growth factor beta receptor II. (TIF) [file pbio.2005907.s003.tif]

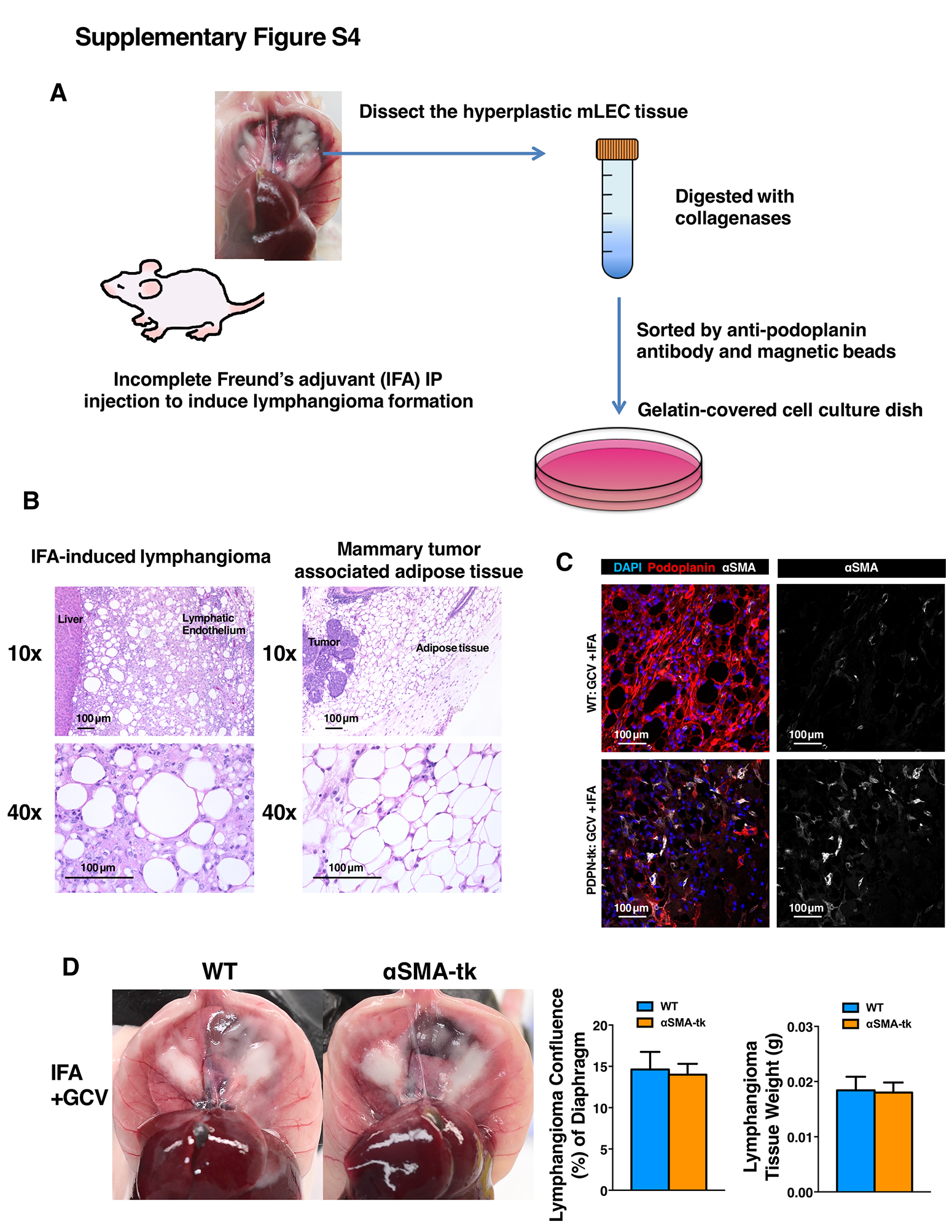

Supplement: S4 Fig — (A) Schematic showing the procedure for the isolation and establishment of mouse primary LEC culture from IFA-induced lymphangioma. (B) The representative images comparing the histology of IFA-induced lymphangioma tissue at the surface of liver and the adipose tissue adjacent to mammary tumor (MMTV-PyMT). Scale bars, 100 μm. (C) Immunofluorescence staining of PDPN (red) and αSMA (white) on the IFA-induced lymphangioma tissues from PDPN-tk and WT mice. Scale bars, 100 μm. (D) The IFA-induced lymphangioma formation was not affected in GCV-treated αSMA-tk mice as compared with GCV-treated WT mice (n = 5 mice per group). Data are represented as mean ± SEM. Significance is determined using an unpaired two-tailed Student t test. The underlying data can be found in S1 Data. αSMA, α-smooth muscle actin; GCV, ganciclovir; IFA, incomplete Freund’s adjuvant; LEC, lymphatic endothelial cell; MMTV-PyMT, mouse mammary tumor virus–polyoma middle tumor antigen; NS, not significant; PDPN, podoplanin; tk, thymidine kinase; WT, wild type. (TIF) [file pbio.2005907.s004.tif]

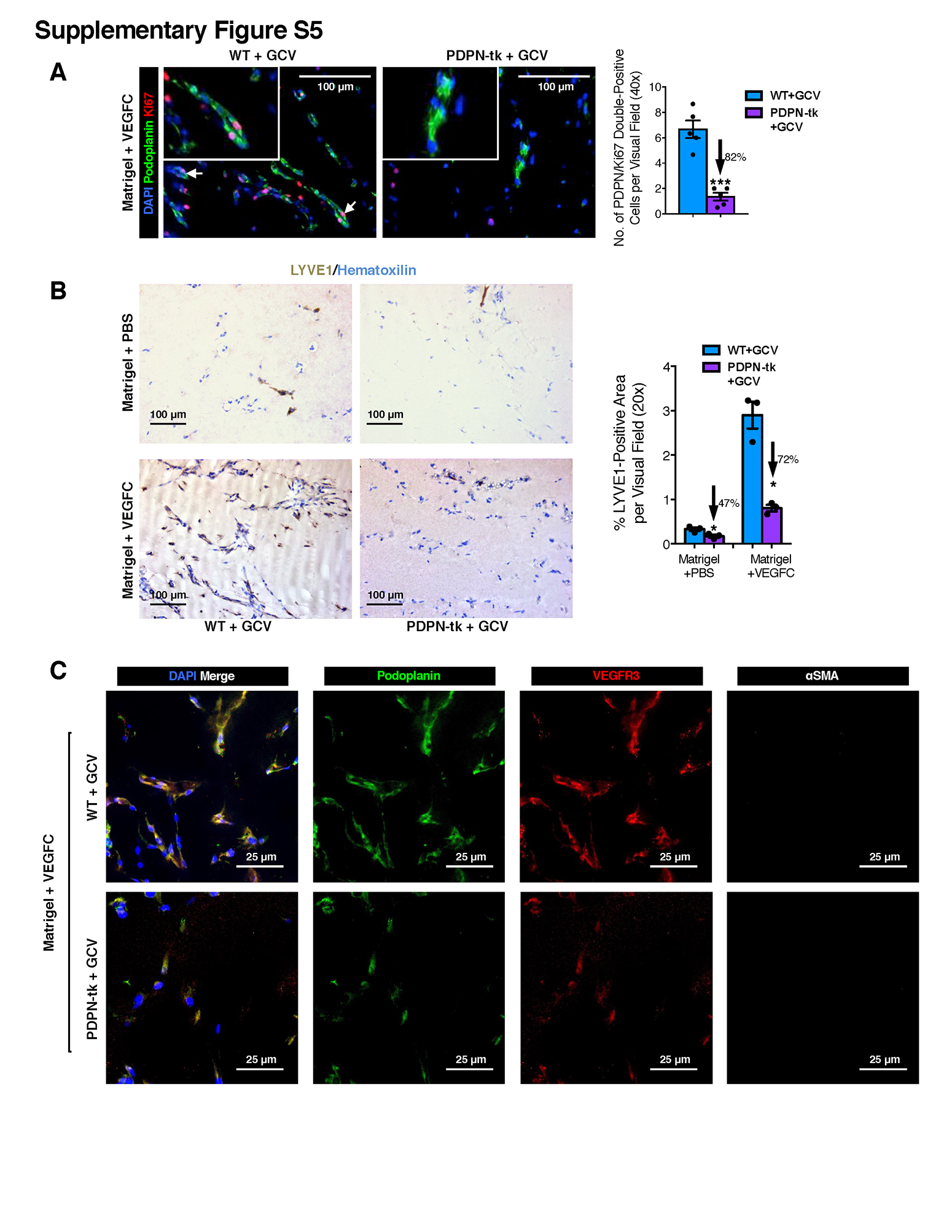

Supplement: S5 Fig — (A–C) Lymphangiogenesis and/or angiogenesis in subcutaneously implanted matrigel plugs (growth-factor reduced, supplied with VEGF-C or PBS) from PDPN-tk or WT mice (n = 5 mice per group). The proliferation status of LECs was examined by Ki67 and PDPN immunofluorescence staining (A). Overall lymphatic vessel density was also evaluated by IHC staining for LYVE1 (B). Scale bars (A–B), 100 μm. The cell populations within the matrigel plugs were further examined by PDPN, VEGFR3, and αSMA immunofluorescence staining (C). Scale bars (C), 25 μm. Data are represented as mean ± SEM. Significance is determined using an unpaired two-tailed Student t test (*p < 0.05, ***p < 0.001). The underlying data can be found in S1 Data. αSMA, α-smooth muscle actin; IHC, immunohistochemistry; Ki67, cell proliferation antigen Ki-67; LEC, lymphatic endothelial cell; LYVE1, lymphatic vessel endothelial hyaluronan receptor-1; PDPN, podoplanin; tk, thymidine kinase; VEGF-C, vascular endothelial growth factor C; VEGFR3, vascular endothelial growth factor receptor 3; WT, wild type. (TIF) [file pbio.2005907.s005.tif]

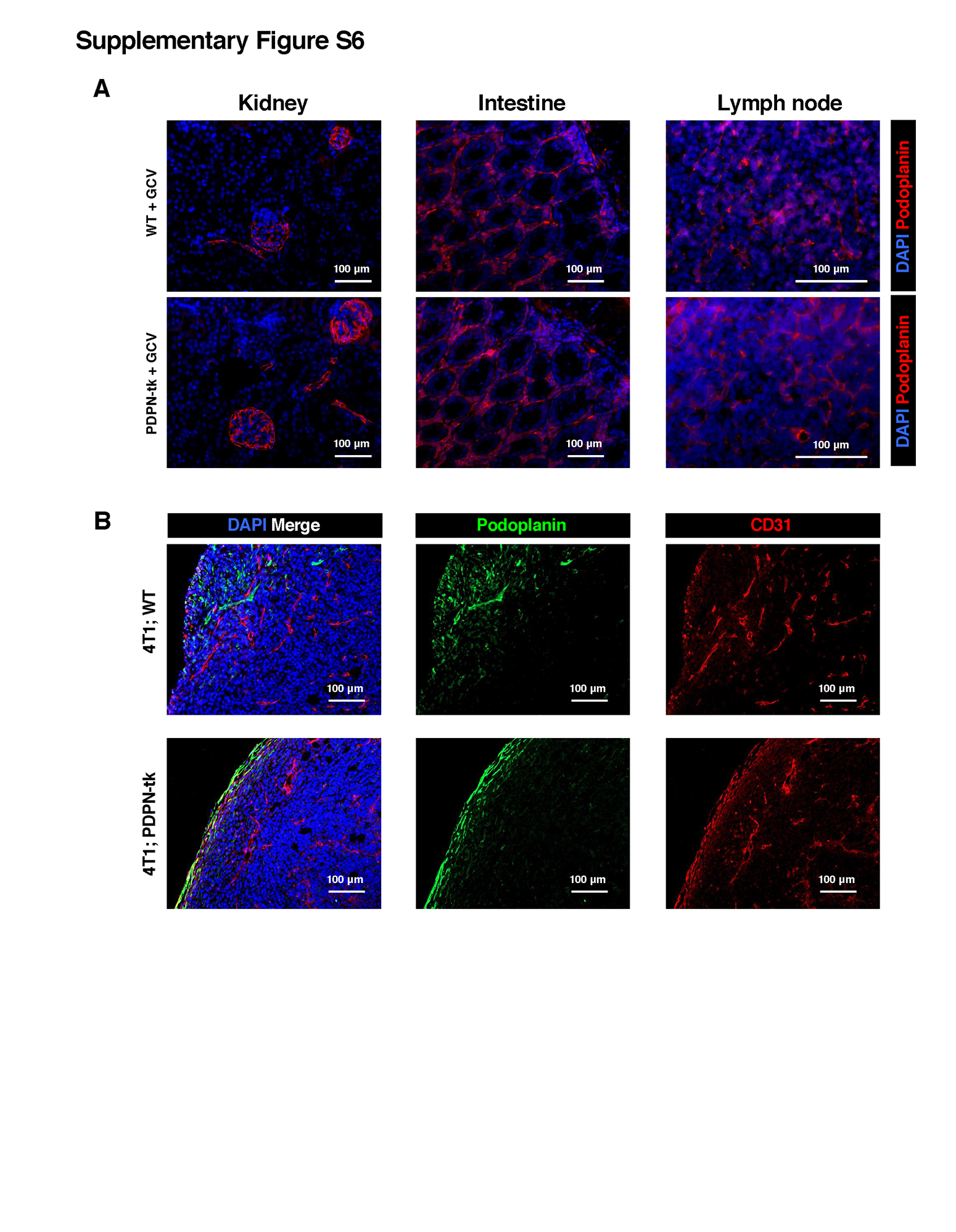

Supplement: S6 Fig — (A) Images showing the PDPN immunofluorescence staining (red) in the kidney, intestine, and LN of GCV-treated WT and PDPN-tk mice. Scale bars, 100 μm. (B) Orthotopic 4T1 mammary tumors in PDPN-tk or WT mice (n = 5 female mice per group) examined for lymphatic vessel density (PDPN staining) and blood vessel density (CD31 staining). These data are also shown in Fig 2C. Scale bar, 100 μm. CD, cluster of differentiation; GCV, ganciclovir; LN, lymph node; PDPN, podoplanin; tk, thymidine kinase; WT, wild type. (TIF) [file pbio.2005907.s006.tif]

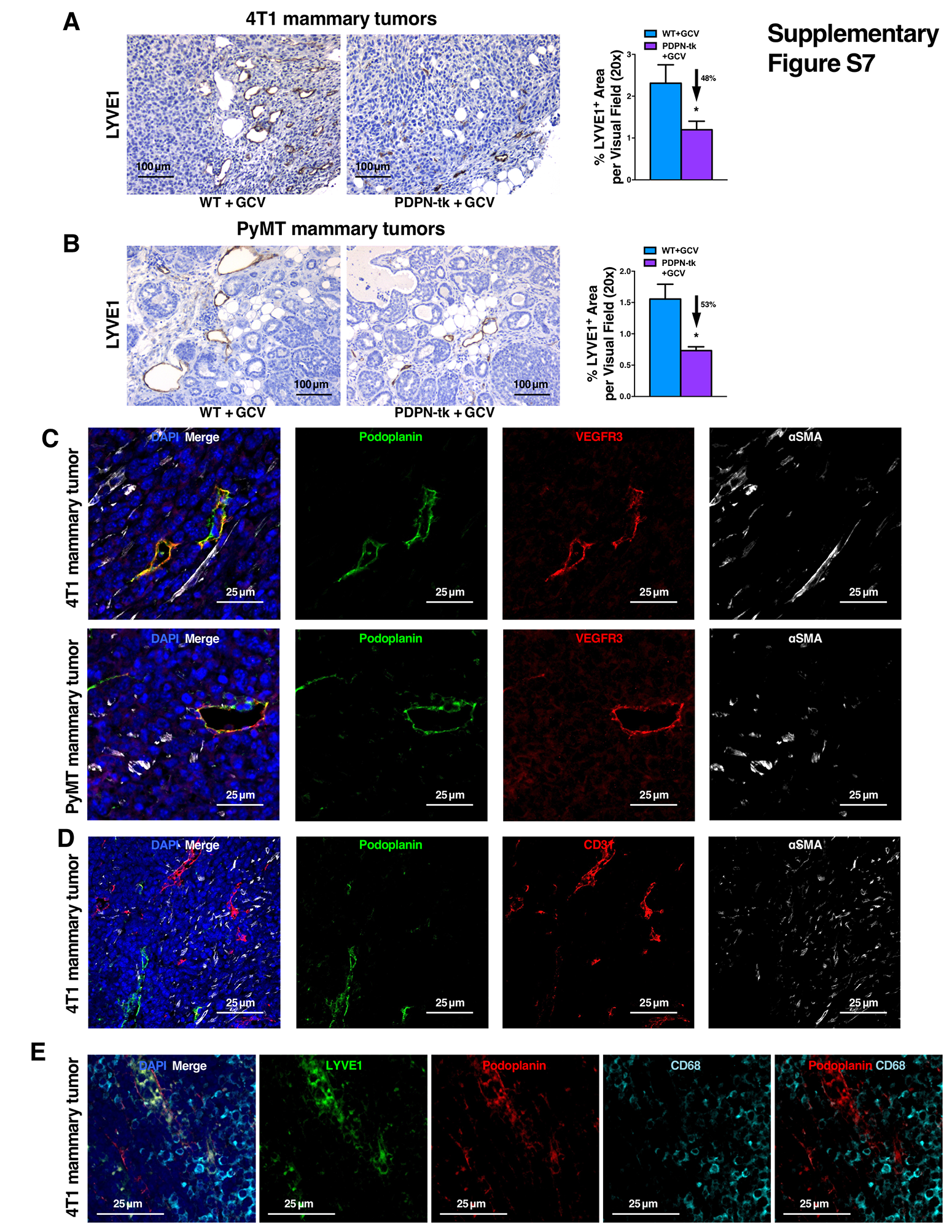

Supplement: S7 Fig — (A and B) Representative images of LYVE1 immunohistochemical staining in 4T1 orthotopic mammary tumors (A) or MMTV-PyMT spontaneous mammary tumors (B) from PDPN-tk or WT female mice (n = 5 per group). Scale bars, 100 μm. Data are represented as mean ± SEM. Significance is determined using an unpaired two-tailed Student t test (*p < 0.05). (C) Representative images of 4T1 and MMTV-PyMT mammary tumors (WT mice) stained for PDPN, VEGFR3, and αSMA. Scale bars, 25 μm. (D) Representative images of 4T1 mammary tumors (WT mice) stained for PDPN, CD31, and αSMA. Scale bars, 25 μm. (E) Representative images of 4T1 mammary tumors stained for PDPN, LYVE1, and CD68. Scale bars, 25 μm. The underlying data can be found in S1 Data. αSMA, α-smooth muscle actin; CD, cluster of differentiation; LYVE1, lymphatic vessel endothelial hyaluronan receptor-1; MMTV-PyMT, mouse mammary tumor virus–polyoma middle tumor antigen; PDPN, podoplanin; tk, thymidine kinase; VEGFR3, vascular endothelial growth factor receptor 3; WT, wild type. (TIF) [file pbio.2005907.s007.tif]

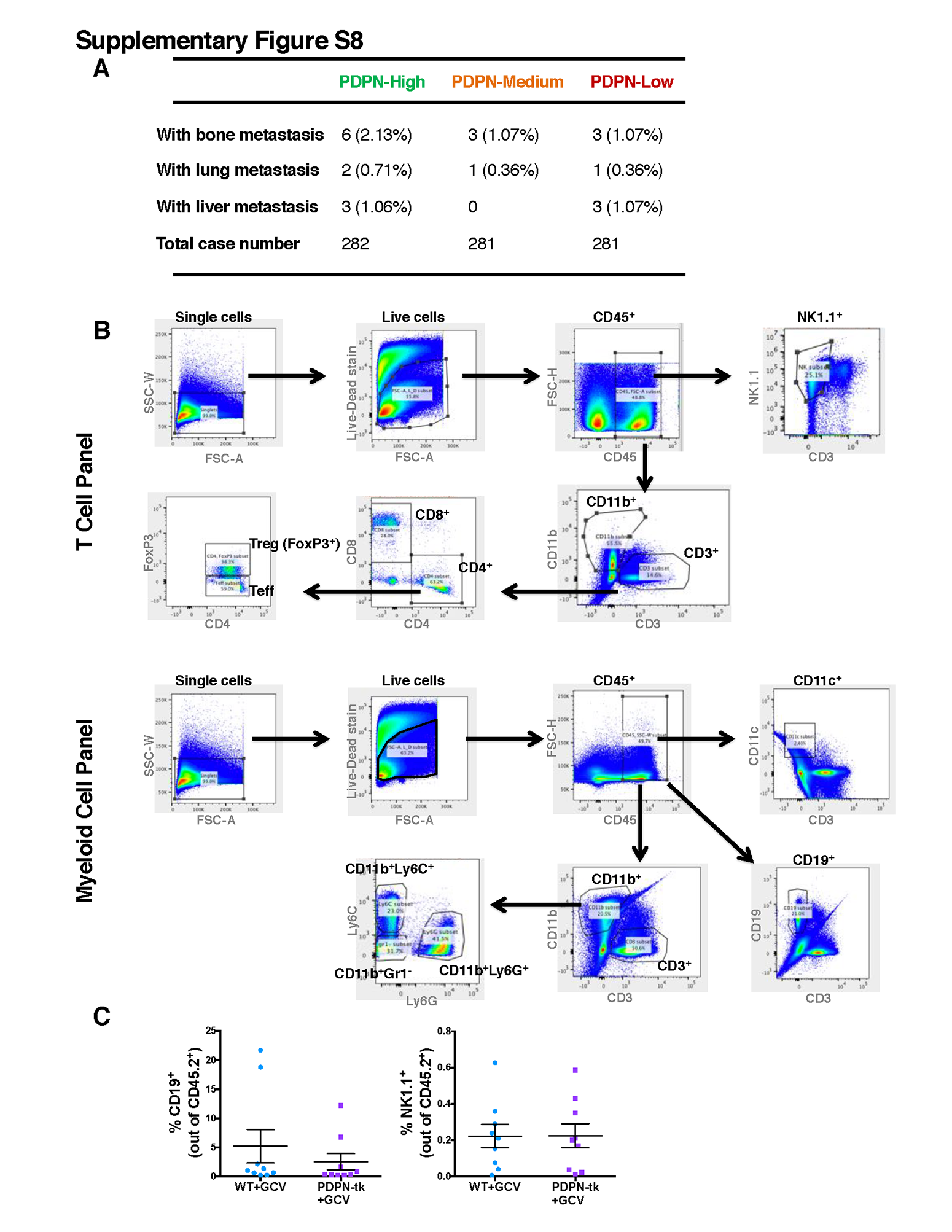

Supplement: S8 Fig — (A) The correlation between PDPN relative mRNA level and distant organ metastasis in 844 breast cancer patients with available data from TCGA breast invasive carcinoma data set. (B) Representative flow cytometry gating strategies used to analyze mammary tumor immune infiltration profile for T cell panel and myeloid cell panel, respectively. (C) Percentages of CD19+ and NK1.1+ cells in 4T1 orthotopic mammary tumors of PDPN-tk or WT mice (n = 9 per group). The underlying data can be found in S1 Data. CD, cluster of differentiation; NK, natural killer; PDPN, podoplanin; TCGA, The Cancer Genome Atlas; WT, wild type. (TIF) [file pbio.2005907.s008.tif]
